# Why Resilience in Health Care Systems is More than Coping with Disasters: Implications for Health Care Policy

**DOI:** 10.1007/s41471-022-00132-0

**Published:** 2022-04-08

**Authors:** Doris A. Behrens, Marion S. Rauner, Margit Sommersguter-Reichmann

**Affiliations:** 1 Department for Economy and Health, University of Continuing Education Krems, Krems/Donau, Austria; 2 Public Health Unit, Aneurin Bevan University Health Board, Caerleon, Wales UK; 3 School of Mathematics, Cardiff University, Cardiff, Wales UK; 4 Department of Business Decisions and Analytics, University of Vienna, Vienna, Austria; 5 Department of Finance, Karl-Franzens University Graz, Graz, Austria

**Keywords:** Resilience Dimensions, Health Care, Interface Management, Behaviour, Systems Thinking, I18, Z18, H12

## Abstract

Health care systems need to be resilient to deal with disasters like the global spread of the Severe Acute Respiratory Syndrome Coronavirus (SARS-CoV-2) on top of serving the changing needs of a multi-morbid, ageing and often dispersed population. This paper identifies, discusses and augments critical dimensions of resilience retrieved from the academic literature. It pulls together an integrated concept of resilience characterised by organisational capabilities. Our concept does not focus on the micro-level like most resilience literature in health care but addresses the system level with many stakeholders involved. Distinguishing exogenous shocks to the health care system into adverse events and planned innovations provides the basis for our conclusions and insights. It becomes apparent only when dealing with planned interventions that transformative capabilities are indispensable to cope with sudden increases in health care pressures. Due to the current focus on absorptive and adaptive resilience, organisations over-rely on management capabilities that cannot generate a lasting increase in functionality. Therefore, reducing the resilience discussion to bouncing back from adverse events could deceive organisations into cultivating a suboptimal mix of organisational capabilities lacking transformative capabilities, which pave the way for a structural change that aims at a sustainably higher functionality.

## Introduction

Climate change, a growing number of disasters, political instabilities and economic turmoil leave the global community desperate for systems that can cope. “Resilience” is (again) the term of the hour. Resilience (Latin for rebounding) is, in such-like contexts, usually understood as the ability to “spring or bounce back” after unexpected adversity. The situation should ideally quickly return to a stable, recovered condition of sufficient quality (for the wellbeing of all systems, objects and subjects) without compromising long-term development (European Commission 2016).

Recently, the Severe Acute Respiratory Syndrome Coronavirus (SARS-CoV-2) has hit the world. The SARS-CoV‑2 pandemic has since depleted health care systems and exhausted their workforces (e.g., Heath *et al.* 2020). Worldwide, improving health care systems’ resilience has dramatically moved up on the agenda (e.g., Haldane and Morgan 2021; Haldane *et al.* 2021; Iyengar *et al.* 2020; Ridde *et al.* 2021). Moreover, Haldane and Morgan (2021) argued that the insights generated during the pandemic represent an opportunity to overcome long-standing structural inequalities in the health care sector and make the environment more sustainable (moving towards a higher level of resilience). Iyengar *et al.* (2020) highlighted further learning opportunities in personal hygiene, reinforcement of infection control measures and the potential of a quicker diffusion of telemedicine.

Despite a univocal call for resilience in health care, the lack of agreement and vision concerning what it actually stands for slows down the joint efforts of policymakers, health care managers, clinicians and patients to build health care systems characterised by resilience. Therefore, it is vital to clarify the concept’s meaning in health care, make it tangible for the people shaping health care by what they do (not only systems engineers), and guide designing resilient systems for practice.

In this paper, resilience should be understood as the capability “to succeed under varying conditions” (Hollnagel 2011), not merely as the capacity to absorb an adverse event (e.g., Lebel *et al.* 2006; Blanchet *et al.* 2017). The paper aims to contribute to the resilience literature applied to the health care sector, keeping (health care) managers in mind. It synthesises and augments essential concepts from a vast body of literature to identify five critical dimensions of resilience in health care and presents an innovative, integrated resilience path concept. These paths are characterised by organisational core capabilities available within a health care system and evaluated for two distinctively different events—adverse event and planned intervention.

After a brief literature review in Sect. 2, Sect. 3 discusses five critical resilience dimensions based on systems thinking principles (de Savigny and Taghreed 2009; Blanchet *et al.* 2017; Wolstenholme and McKelvie 2019) in the context of the event type. Regarding the latter, we distinguish between adverse events and planned interventions. We add planned interventions to the resilience concept because they are frequently overlooked in practice, although they teach us what it takes to recover from adverse events successfully in the long run. The resilience dimensions are mostly illustrated with examples from British National Health Service (NHS) daily practice (due to the professional background of one of the authors). The paper closes with Sect. 4, which brings together our main conclusions and explains barriers to picking up a transformative resilience path in health care.

## Application Areas and Methodologies to Investigate Resilience

Numerous books, review papers and reports have sought to discuss resilience within the framework of a wide range of application areas. Among these are psychology, sociology, ecology, urban planning, disaster management, business administration and health care (e.g., Bhamra *et al.* 2011; Hollnagel *et al.* 2006; Hollnagel 2011; Karidi *et al.* 2017; Korber and McNaughton 2018; Martin-Breen and Anderies 2011; Tusaie and Dyer 2004; Welsh 2014; Wiig and Fahlbruch 2019). “Resilience” was initially used as a descriptive-analytical term and then transformed into a more general system analysis concept (Welsh 2014). However, several problems emerged when adapting the original psycho-social concept elsewhere. This is reflected by the spectrum of methodologies when investigating resilience. Depending on the application area (e.g., Meerow and Newell 2015; Kochan and Nowicki 2018; Sepúlveda Estay *et al.* 2020), methodologies range from empirical studies (e.g., case studies, surveys, field studies) to analytical research. Kochan and Nowicki (2018) showed, for example, that multi-criteria decision analysis, network modelling and simulation dominate the analysis of supply chain resilience. When discussing industrial ecology resilience (Meerow and Newell 2015), life cycle assessment, material flow analysis and input-output analysis prevail. Regarding resilience within the context of cyber-security (Sepúlveda Estay *et al.* 2020), systems architecture and algorithms to enhance the security of information systems play a vital role. System dynamics proved valuable insight for understanding complex systems (Brailsford *et al.* 2004; Lane *et al.* 2000; Wolstenholme and McKelvie 2019), with game theory and agency theory assisting in investigating stakeholder behaviour (e.g., Djulbegovic *et al.* 2015; Windle 2011).

In addition, resilience relates to concepts of “sustainability”. Originally, sustainability referred to the relationship between humans and nature, emphasising the improvement of human life and the conservation of natural resources (i.e., sustainable development), as outlined in the 1980 World Conservation Strategy (Giovannoni and Fabietti 2013). Sustainability aimed at environmental systems prevailing in the long run. Since its early days, the sustainability concept has evolved into a multi-disciplinary approach by incorporating interconnections among environmental, economic and social agendas (see, e.g., the Rio +20 United Nations Conference on Sustainable Development in 2012). Currently, integrated sustainability concepts are on the rise. They incorporate governance, strategy and business models alongside measurement and reporting systems. For example, the concept of “dynamic sustainability” emphasises the system’s ability to react to environmental changes to survive under changing conditions (Flessa and Meissner 2019). In contrast, the concept of “functional sustainability” addresses a system’s ability to provide services and produce goods (Flessa and Meissner 2019). Depending on current circumstances or future needs, a system can maintain or change its structure, leading to the concept of “structural sustainability” (Flessa and Meissner 2019). The sustainability context hosts these diverse approaches and embraces tensions due to apparent trade-offs. While we acknowledge the nexus between resilience and sustainability, we seek to contribute to a richer resilience concept in health care that does not reduce resilience to merely bouncing back after an adverse event.

Concepts from quality management (Oakland *et al.* 2020) have contributed, furthermore, to enhancing system resilience, such as the Deming cycle (plan-do-check-act). For example, Hollnagel (2011) suggested a Resilience Analysis Grid to help general organisations develop an overall resilience profile and overcome deficiencies. Looking at the health care sector, Anderson *et al.* (2016, 2020) used a Concepts for Applying Resilience Engineering (CARE) model to improve health care quality (by developing and testing suitable interventions). Meyer *et al.* (2020) developed a Health System Resilience Checklist for measuring capacities, capabilities and processes to foster resilience when facing natural hazards and infectious disease outbreaks. To investigate the SARS-CoV‑2 resilience crisis in Europe, Aristodemou *et al.* (2021) reported on an After-Action Review (AAR) study. It aimed to help health care policymakers to react more swiftly/decisively and coordinate a crisis across countries using three indices: 1) the preparedness of countries’ health systems to deal with a potential health shock (Health System Preparedness Index, HSPI), 2) the strictness of confinement measures (Government Response Confinement Index, GRCI) and 3) the expected socio-economic effects of these measures (Socio-Economic Impact Index, SEII).

Furthermore, risk management concepts are strongly related to the resilience concept (e.g., Merna and Al-Thani 2012; Hopkin 2018). Risk management standards are valuable concepts for organisations to enhance their resilience, as outlined Haddad and Laghzaoui (2020). The “ISO (International Organization for Standardization) 31000—Risk Management—Guidelines” represent a crucial standard, “*which* *provides a level of reassurance in terms of economic resilience, professional reputation and environmental and safety outcomes*” (ISO 2022a). Currently, the “ISO 31050—Guidance for Managing Emerging Risks to Enhance Resilience” is under development to cope with emerging risks and uncertainties (ISO 2022b), such as the SARS-CoV‑2 pandemic and improve the resilience of the health care infrastructure (Jovanović *et al.* 2020).

The health care system is part of the critical infrastructure (on a local, regional, national and even international level). Making health care systems resilient is therefore of utmost societal importance. Recent literature reviews summarise several critical issues (e.g., Abimbola *et al.* 2019; Atkinson 2020; Barasa *et al.* 2017; Berg and Aase 2019; Biddle *et al.* 2020; Blanchet *et al.* 2017; Ellis *et al.* 2019; Fallah-Aliabadi *et al.* 2020; Fridell *et al.* 2020; Hanefeld *et al.* 2018; Iflaifel *et al.* 2020; Turenne *et al.* 2019; Wahedi *et al.* 2019). For example, Hanefeld *et al.* (2018) illustratively investigated the health care system response to adverse events such as an Ebola outbreak, financial crisis and climate change. They also examined how they impact selected health care functions (health information systems, funding/financing mechanisms, health workforce). The authors concluded with an outline of the lessons learnt and proposed strategies to enhance system resilience. More recently, improving health care resilience in the context of the SARS-Cov‑2 crisis focused on improving risk assessment approaches and drawing on experiences from around the world (see, e.g., Jovanović *et al.* 2020; Trump and Linkov 2020; Haldane *et al.* 2021).

Another core component of health care resilience is supply chain resilience by maintaining customer satisfaction, enhancing the efficiency of responding to disruptions and reconstructing the supply chain (e.g., Han *et al.* 2020; Hosseini *et al.* 2019; Kamalahmadi and Parast 2016; Katsaliaki *et al.* 2021; Kochan and Nowicki 2018).

Finally, we acknowledge that the health care system critically depends on the personal resilience of all people involved, i.e., health care professionals and patients (e.g., Tusaie and Dyer 2004; Windle 2011; Zhang *et al.* 2022). As personal resilience impacts health care resilience, human resources management in companies (e.g., Lefebvre *et al.* 2020) and health care institutions (e.g., Hart *et al.* 2014; Jackson *et al.* 2007; Labrague 2021; Palacio Gonzalez *et al.* 2020; Robertson *et al.* 2016) introduced resilience concepts to nurture the workforce’s ability to cope with stress and adversity (both in the working and private environments).

## Critical Dimensions of Resilience

Researchers, clinicians and policymakers agree that health care systems need to be resilient—not only to withstand natural disasters, disease outbreaks and climate change consequences. Also, health care systems must serve the changing needs of an ageing and often dispersed population with increasing mental health issues (World Health Organization (WHO) 2014, 2018, 2020). Still, in health care management’s daily practice, we often observe the absence of a (shared) understanding of what it takes to make the health care system resilient. The reason is that the term resilience is used interchangeably in different research fields—but without field-specific definitions (Conz and Magnani 2020; Martin-Breen and Anderies 2011).

This section aims at identifying and discussing dimensions vital to pin down a newly proposed resilience concept for the health care sector based on systems thinking principles (Blanchet *et al.* 2017; Rutherford 2019; de Savigny and Taghreed 2009; Senge 1990; Wolstenholme and McKelvie 2019). Specifically, we rely on distinguishing between an adverse event and a planned intervention. In both cases, planned interventions (e.g., health care reforms) and adverse events (e.g., the SARS-COV‑2 pandemic), the same people (as part of the system) and processes “respond” to the event, most likely along different resilience paths. Still, our discussion focuses on the system level, i.e., the whole system’s response to an event (and not the micro-level).

**Fig. 1:**
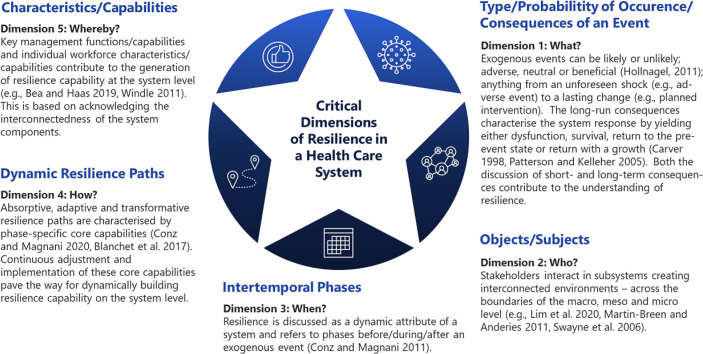
Critical dimensions for building a resilient health care system

Fig. 1 illustrates five critical resilience dimensions retrieved from in-depth literature research. We will elaborate on these aspects, dimension by dimension (cf. Sects. 3.1–3.5): 1) type/probability of occurrence/consequences of an event, 2) objects/subjects, 3) intertemporal phases, 4) dynamic resilience paths and 5) characteristics/capabilities.

### First Resilience Dimension: Type/Probability of Occurrence/Consequences of an Event

**Fig. 2:**
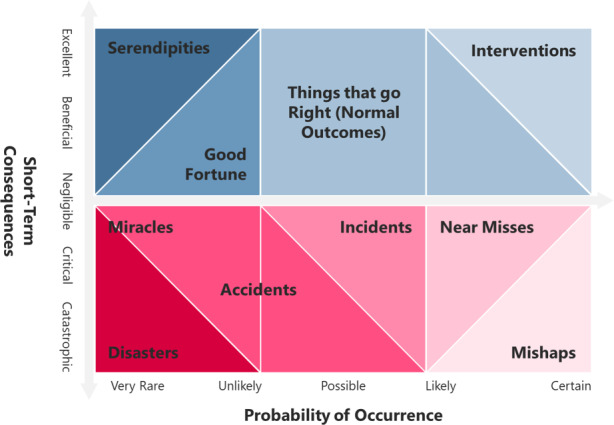
Resilience dimension 1 : characterisation of exogenous events in terms of probability of occurrence and short-term consequences (adapted from Hollnagel 2011)

The first resilience dimension encompasses an exogenous event’s type/probability of occurrence and consequences (see Fig. 2). Traditionally, 1) the *extent of the short-term consequences* (negligible, marginal, critical, catastrophic) and 2) the *probability of occurrence* (rare, unlikely, possible, likely, certain) describe the risk of an event and its type (see, e.g., Hollnagel 2011). The first resilience dimension takes stock of all possible types of events (regardless of the long-run consequences) and categorises them by their short-run consequences. Sect. 3.1.1 concentrates on adverse events, like the SARS-CoV‑2 pandemic, while Sect. 3.1.2 emphasises planned interventions. Sect. 3.1.3 concludes with policy implications for the definition of resilience.

#### Adverse Events

We do not want to limit our discussion to adverse events—but, typically, we have rare catastrophic events in mind when discussing resilience (positioned in the lower left-hand corner in Fig. 2). An example of such a “disaster” was Hurricane Katrina, leaving most of New Orleans inundated for weeks in the summer of 2005. Disasters with minor or no negative (short-term) consequences are categorised as “miracles”. For example, when a flock of birds had hit both engines of U.S. Airways Flight 1549 only minutes after taking off from LaGuardia Airport (New York City), no human lives were lost because the pilots skilfully landed the aircraft in the Hudson River. “Accidents” and “incidents” are adverse events happening more frequently (and thus perceived as more likely) than disasters (e.g., an unplanned interruption of emergency services due to an unexpected capacity constraint presenting a severe threat to the health of the community). “Near misses” (positioned on the lower right-hand side in Fig. 2) are adverse events that happen frequently (e.g., breaching compliance with the British “4-hour target”, which aims at commencing treatment of every Emergency Department (ED) patient within 240 min upon arrival). Hollnagel (2011) describes “near misses” as usually benign but potentially implying severe negative consequences (which rarely happen because these events otherwise would have been eliminated). “Mishaps” are “near misses” with serious outcomes such as spreading hospital germs.

We believe that it takes both an adverse event’s short-term and long-term consequences to discuss resilience in health care in a meaningful way. While the short-term consequences (see Fig. 2) describe how intensely an adverse event strikes (parts of) a system, the long-run outcome identifies how well the more comprehensive system responds. For example, at the onset of the first SARS-CoV‑2 wave in early 2020 (when elective hospital services nearly evaporated), 3097 NHS patients in England waited for more than a year to commence specialist-led elective care (NHS England and NHS Improvement 2020). A year into the pandemic (by the end of April 2021), the number of UK patients waiting more than 52 weeks has increased to 385,490—with the overall waiting list for NHS surgery having surpassed the five-million mark (NHS England 2021b). Elective hospital services may be going back to their pre-event levels of service delivery. However, the health care system will not follow suit. The backlog in demand (causing long waits) and the follow-up effects of delayed diagnoses and treatment (causing even longer waits and additional deaths) are likely to be a feature of the British NHS for several years at least. Also, in other health care systems than the British NHS, feedback and unintended consequences will determine the long-term system functioning (yielding dysfunction, reintegration with a loss, reintegration back to the pre-event status or a reintegration leading to growth; Carver 1998, Patterson and Kelleher 2005) of patient diagnosing, treatment and care.

#### Planned Interventions

Hollnagel (2011) already added the potential of favourable outcomes to the characterisation of an adverse event (see Fig. 2). The latter he calls “serendipities” or “good fortune” if the event was improbable (e.g., the positive consequences of the time commuters salvaged due to the pandemic-induced home-based work mode). By including planned intervention, we seek to highlight another element when discussing exogenous events (in addition to including the event’s long-run consequences) (see the upper right-hand corner in Fig. 2).

In the context of an adverse event, we usually focus on absorptive resilience (to “take the hit” and bounce back). Occasionally, we also observe efforts towards adaptive resilience (should recovery happen by process change and reallocation of inputs). The organisational capabilities to allow adaptive resilience would also lend themselves to deal with (incremental) process improvement. There is one type of resilience, which we hardly ever observe; health care organisations seldom recover from an adverse event through transformative resilience, which alludes to structural changes (alongside process adaptation).

The indispensability of transformative resilience in health care only becomes apparent in the context of planned interventions. Transformative resilience aims at a structural change to achieve higher functionality of the health care system instead of simply absorbing adverse effects and adapting processes to restore pre-shock levels gradually. Analysing the system response to a planned intervention, thus, teaches us that the focus on absorption and adaptation in the context of adverse events is short-sighted because we have to cultivate transformative capabilities to succeed in the long run. It is precisely these transformative capabilities that allow us to achieve greater functionality for both types of events (and that are treated like footnotes within the resilience discussion).

#### Policy Implications for the Definition of Resilience

To review the short- and long-term consequences of exogenous events, it is necessary to re-examine the popular understanding of resilience. If we continued to reduce resilience to merely the ability to bounce back after an exogenous shock, we would *de facto* suggest that highly resilient systems are unlikely to improve (because all they can do is absorb what comes from outside the system). To ensure that a system can cope with stress *and* reinforce intended positive consequences (of interventions or response to an adverse event), we believe that resilience should instead relate to the capability “to succeed under varying conditions” (Hollnagel 2011). This brings forward the discussion of resilience dimension 2.

### Second Resilience Dimension: Objects/Subjects

Depending on the type/probability of occurrence, and consequences of an exogenous event in the health care system (first dimension), all objects and subjects involved (and their aims and priorities) need to be identified in a subsequent step (second dimension). Thus, the second resilience dimension pertains to taking stock of the stakeholders in the health care system, who they are, how they interact, and what they want (Lim *et al.* 2020; Martin-Breen and Anderies 2011). Learning about these relationships and appreciating the multiple perspectives supports the engineering of adequate responses to exogenous events at the system level (Rutherford 2019; Senge 1990; Sterman 2000; Wolstenholme and McKelvie 2019).

**Fig. 3:**
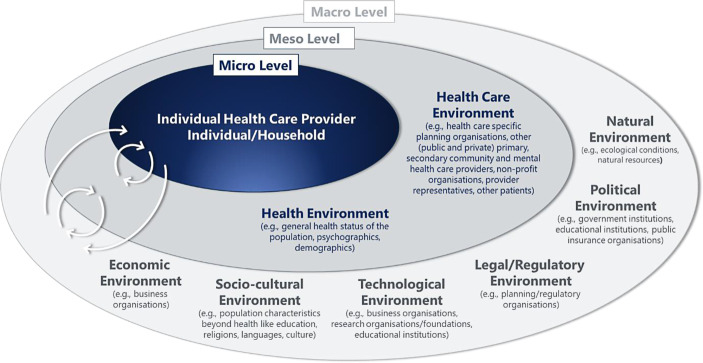
Resilience dimension 2: objects/subjects in an interconnected system (developed based on Lim et al. 2020; Martin-Breen and Anderies 2011; Swayne et al. 2008 and general resilience review literature)

When seeking insight into the health system’s behaviour (in Fig. 3, positioned at the meso level) or the fate of a single health care organisation (at the micro level), we find that the list of stakeholders to include is quite exhaustive. It contains (other departments of) primary, secondary, tertiary, quaternary, community, and mental health care providers, (other) provider representatives, (other) health care staff, and (other) patients, to mention but a few. Furthermore, the health care system impacts the health environment and *vice versa*. Both health care and the general level of health operate within a broader context (indicated by the macro level in Fig. 3). It incorporates six main contextual domains: 1) natural, 2) political, 3) legal/regulatory, 4) technological, 5) socio-cultural and 6) economic environments (Swayne *et al.* 2008). Essential macro-level stakeholders comprise, for example, government institutions, business organisations, educational institutions, research organisations/foundations and religious institutions. All of these are interconnected (cf. Blanchet *et al.* 2017). Stakeholder engagement is vital—especially for a planned intervention and a minor adverse event—for which aims must be skilfully aligned to reach maximum impact.

Berg and Aase (2019) found that resilience studies focused almost exclusively on the micro level (e.g., frontline clinical staff or hospitals/institutions), ignoring the broader context. The latter elides the fact that a single debilitated system component (e.g., an overburdened emergency department) or a single neglected link between subsystems (e.g., a missing protocol governing the transfer of patient notes with the patient from secondary to primary care) can impair the operational capability of the health care system.

To illustrate how stakeholders and their interconnectedness affect the health care system’s capability to overcome an adverse event, let us think of virtual clinics for a moment. During the first wave of the SARS-CoV‑2 pandemic in 2020, telehealth potentially enabled primary health care providers to absorb (some of) the pandemic-induced shock. Wherever the implementation was successful (e.g., Welsh Government 2021), it brought together the technological prerequisites (often existing upfront but put on hold for legal reasons), the overdue legal permission (due to the sensitive nature of the patient-clinician interaction), the political support (releasing vital funding) and the general practitioners’ willingness to offer this innovative service approach. It was based on the population’s health care needs and—depending on the socio-cultural context—on the patients’ willingness and ability to accept the virtual service offer. In this context, the shared challenge of SARS-CoV‑2 aligned multiple stakeholder perspectives and united them through a shared “enemy”.

A significant adverse event like the SARS-CoV‑2 pandemic unites stakeholders. We justify this statement with the emergency of the following causal chains over the past 18 months. In networks of organisations (that collaborated due to SARS-CoV‑2), many aims boiled down to one. This mostly translated into identical goals, especially when the collaboration was built around an overarching (specific) purpose, e.g., testing or tracing contacts. Then, these interconnected organisations started to share priorities. The resulting synchronisation of the entire aim-goal-purpose framework of the involved stakeholders produced remarkable success stories. The Welsh Test Trace Protect services are such-like examples (Audit Wales 2021; Technical Advisory Group 2021), especially the service for the Gwent region in the southeast of Wales. An exemplary network of diverse organisations brought together national and local expertise. The service consisted of parts of the NHS and many local authorities, including so-called environmental health officers (before dealing with local outbreaks of measles, chickenpox and other diseases). Staff were recruited from the private and third sectors and the wider public sectors for managerial work and local call centres. The service had diversity written all over—the only exception being the aim, goal and purpose of the collaboration. These three were crystal clear to all staff, hugely engaging and united the organisations and synchronised processes.

Like in the case of responding to global adverse events (see e.g., Alexander 2002), a planned intervention (but also the response to a local adverse event) is most effective when all stakeholders align their aims, goals and priorities due to an overall purpose (e.g., Neville *et al.* 2016; Rauner *et al.* 2018). Clarity of purpose is mandatory; a clear *shared* purpose is even superior. In the absence of an external uniting force, aligning aims, goals and priorities is challenging work, and often there is no success in uniting stakeholders. In this case, it would be wise to investigate the limits of what can be achieved before launching an intervention. Otherwise, the health care system will absorb the well-intended efforts.

When we consider a minor (local) adverse event (e.g., a major accident on a motorway, a landslide, a flood), the alignment of aims and goals may be jeopardised. When setting up response plans, these (soft and fluffy) factors are as important for planning as allocating resources according to emergency protocols and training. Especially for major cross-border emergencies such as pandemics, these emergency protocols and trainings should be set up with a clear joint aim in mind (see e.g., Neville *et al.* 2016; Rauner *et al.* 2018; Steen *et al.* 2017).

### Third Resilience Dimension: Intertemporal Phases

**Fig. 4:**
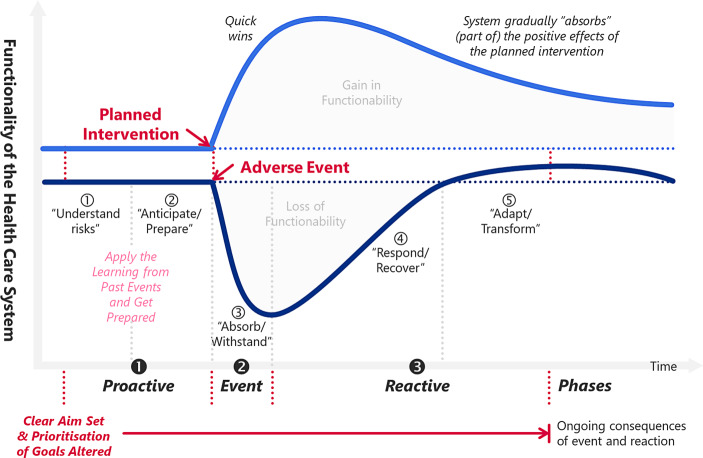
Resilience dimension 3: Categorisation of current situation relative to a planned intervention/an adverse event, split into phases (graph adapted from Jovanović et al. 2020)

Resilience dimension 3 refers to categorising the current situation relative to the timing of the exogenous event (cf. Fig. 1). For example, for adverse events, Jovanović *et al.* (2020) use five phases following the structure of the SmartResilience project (EU-VRi 2022). The related categorisation into phases is displayed in the lower part of Fig. 4 to provide context (the terms in quotation marks are introduced by Jovanović *et al.* (2020)).

For adverse events, we take a generic approach and (more or less) merge Jovanović *et al.* (2020)’s phases devoted to *understanding the risks* and *anticipating and preparing* for the event (see Fig. 4). Also, we do not differentiate between the *response* until the system’s critical functionality is regained after an exogenous adverse event and the (potential) *transformation* that follows the response phase (see the lower part of Fig. 4). The latter is because the long-run state of the system and the path the system takes to get there critically depend on how we respond in the first place. Transformation starts a lot earlier than at the moment of regaining pre-shock levels. Moreover, *response* and *transformation* are intertwined, making drawing a line in the sand operationally tricky. Therefore, we follow (Conz and Magnani 2020) and split time into three chunks:

1. a proactive phase (before the event)
2. an event phase (taking the event)
3. a reactive phase (after the event) (see the bottom part of Fig. 4).

The proactive phase starts when a health care system/organisation alters its aim(s) and prioritises different goals concerning an anticipated or hypothetical future event (see Fig. 4). We define the endpoint of the reactive phase as when the aim/prioritisation of goals focuses on something different than the exogenous adverse event. This endpoint usually does not coincide with regaining re-shock functionality or the realisation of the event’s long-run consequences.

If we think of the first wave of the SARS-CoV‑2 pandemic, the news from China kicked off health care planning for different scenarios in mainland Europe and the UK in early February 2020 (WHO 2022). When SARS-CoV‑2 spread in Northern Italy like a wildfire, the preparatory actions took momentum. Health care organisations rapidly changed their priorities with the precise aim of not letting people die due to capacity constraints. This marked the beginning of the proactive phase. The WHO’s reports from late February/early March 2020 made it crystal clear to get (operationally) ready for the expected increase in workload (WHO 2021). Across Europe, health care providers cancelled elective procedures, stockpiled oxygen and personal protective equipment, and started to turn sports stadiums, leisure centres and exhibition halls into field hospitals. By mid-March to early April 2020, most European countries reached the beginning of the event phase and absorbed a massive demand shock. Case numbers increased exponentially, and with a three-week delay, the demand for Intensive Care Unit (ICU) beds; all hands were on deck; all resources focused on dealing with the inevitable. The reactive phase began when health care providers started to respond in a more planned manner, redeployed resources (e.g., beds, workforce, ventilators, disposable equipment, pharmaceuticals) and utilised innovations (e.g., telehealth).

The onset of the proactive phases regarding the second wave varied across countries, depending on how hard the initial wave had hit the respective regions. Those health care systems whose capacities had been breached during wave 1 started planning for the second wave in late May/early June 2020 (others followed later). They went into a loop and (re)entered the proactive phase of the second SARS-CoV‑2-wave. The onset of the event phase hugely depended on public health measures implemented by governments and the populations’ compliance with SARS-CoV‑2-measures. In combination with health and public services “doing the right things” (not only “doing things right”), these measures allowed even countries with a small hospital capacity per 100,000 population (like the UK) to (mostly) get through the second SARS-CoV‑2-wave without triaging SARS-CoV‑2-patients.

Due to the differences in vaccination uptake and the timing of its roll-out, it is impossible to describe the phases of the third wave generically (not even across Europe). One country that quickly rolled out vaccination strategies was Israel with about 80% of the eligible population having received three doses by the end of 2021 (Burki 2022). A fourth dose is currently discussed to be administered to health care staff and people older than 60 years because Israel’s hospitalisation rates have been on the rise since January 2022.

The electronic patient records implemented years ago build the bridge to a planned intervention (see, e.g., Safran 2000; Thiru *et al.* 2003). Electronic patient records aimed to improve treatment quality and patient safety and increase the functionality of the health care system by facilitating patients’ and service providers’ access to relevant health data (e.g., clinical discharge reports, lab and X‑ray data, prescription and non-prescription drugs). European nations are at different maturity levels when it comes to electronic patient records (see, e.g., European Observatory on Health Systems and Policies 2022). To pick one example, in mid-2020, Austria experienced electronic health records helpful in paving the way for swiftly implementing e‑medication and, later on, electronic vaccination certificates (ELGA GmbH 2022). At that time, the (planned intervention’s) event and the reactive phases were completed. However, the SARS-CoV‑2 pandemic revealed a lack of foresight in transformative capabilities, particularly data and interface management. At the end of the third wave, missing data and missing links between vaccination and hospitalisation data made it challenging to respond to the pandemic in an evidence-based manner (Kada *et al.* 20.08.^2021^). In this case, it was more than just unintended long-run consequences revealed substantially past the intervention date. It was a “baptism of fire” when an adverse event hit the health care system post-intervention. The lessons learnt will help to transform the health care system and prepare for other (local and global) adverse events.

### Fourth Resilience Dimension: Dynamic Resilience Paths

Conz and Magnani (2020) created two dynamic resilience paths (resilience dimension 4) for situations where companies cope with disturbance: the absorptive path (Sect. 3.4.1) and the adaptive path (Sect. 3.4.2). While the absorptive path lends itself only to dealing with adverse events, the adaptive path is also suitable for more minor planned interventions concentrating on incremental and sustainable measures at the subsystem level. Therefore, we additionally draw on a concept of Blanchet *et al.* (2017) and integrate a so-called transformative resilience path (Sect. 3.4.3) into Conz and Magnani (2020)’s business-oriented framework. In so doing, we can discuss planned intervention in a health care system—alongside bouncing back after a shock—in a resilience context.

**Fig. 5:**
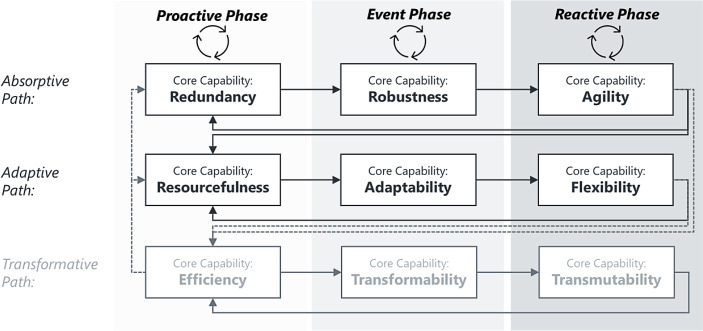
Resilience dimensions 4: dynamic resilience paths evolving through phases (adapted by the authors based on Conz and Magnani 2020; Blanchet et al. 2017 and general resilience review literature)

Within this integrated framework, we refer to a *path* as a dynamic process of gradually building resilience through acquiring and adjusting **organisational core capabilities** within the corresponding health care system (cf. Fig. 5). The core capabilities, essential to building absorptive and adaptive resilience, are taken from Conz and Magnani (2020) and displayed in Fig. 5. We derive the transformative core capabilities from the general resilience review literature (e.g., Aburn *et al.* 2016; Iflaifel *et al.* 2020; Korber and McNaughton 2018; Turenne *et al.* 2019; Windle 2011).

The proactive phase’s core capabilities (see Fig. 5) are the precursors of the core capabilities deployed during the event phase. These newly developed capabilities then foster developing reactive core capabilities.

#### The Absorptive Resilience Path

Conz and Magnani (2020) identified ***redundancy, robustness and agility*** as core capabilities when an organisation progresses along the absorptive resilience path (see Fig. 5). Recall that the concept of an absorptive path lends itself only to coping with adverse events.

As outlined in Sect. 3.3, the proactive phase starts when a health care organisation alters its aim(s) and prioritises other goals in anticipation of a hypothetical event (see Figs. 4 and 5). When progressing along the absorptive path, strategically accumulating redundancies is vital to prepare for adverse events on a system level. Strategically creating ***redundancies*** includes filling inventories (equipment or medication), establishing backup teams (clinical, managerial, auxiliary), creating surge capacities concerning hospital beds and operating theatre slots, or setting up field hospitals in anticipation of a SARS-CoV-2-patients surge.

Swift access to the resources not needed in the proactive phase is the prerequisite for achieving operational ***robustness*** (at the onset of an event phase). The latter capability prevents (or reduces) the adversity’s impact without modifying the health care system’s stable pre-event configuration. An example of creating robustness out of redundancy is operating a health care service at a utilisation of less than 100%, where surge capacity enables the service to cope with sudden demand peaks. Note that these demand peaks are unexpected at the time of occurrences but not unexpected on the grand scale of schemes – this is why health systems *strategically* prepare for these demand peaks. The operationalisation of the surge capacity varies, however, among countries. For example, the German health care system exhibits an average hospital bed utilisation of around 77% (Statistisches Bundesamt 2019). Before the SARS-CoV‑2 pandemic took off, the busy British NHS faced a bed utilisation of more than 88% (NHS England 2021a); during winter pressures, the utilisation was consistently around 95% (NHS Providers 2018).

Once a health care system has established robustness, the core capability of ***agility*** emerges in the reactive phase. Imagine an accident on a motorway with injured people requiring emergency treatment. The accident is an adverse event that a local hospital can deal with using the staff on duty (plus on-call backup teams) and the available health care infrastructure. In this case, ample resources and the strategies already in place (in anticipation of a demand shock at some point in time) absorb the shock and generate the capability to respond quickly and effectively (without altering the routines and strategies of the health care system). This applies particularly to the care provided to the injured people after receiving emergency treatment and being admitted from the emergency department to an ICU or hospital ward in the reactive phase. However, changing the pre-event configuration of service delivery may be necessary to deal with an exogenous shock, which takes us to the adaptive resilience path.

#### The Adaptive Resilience Path

***Resourcefulness, adaptability*** and ***flexibility*** characterise the adaptive resilience path (Conz and Magnani 2020). The organisational capability to strategically accumulate and take stock of diversified (physical, human, technological, management and financial) assets and resources (***resourcefulness***) in anticipation of an adverse event or a minor planned intervention enables sustaining the system’s operational capability during the event phase (Pal *et al.* 2014). The core capability that emerges from resourcefulness is ***adaptability*** (i.e., adjusting internal processes, recombining resources and continuously reconstructing the health care system). Adaptability then entails the capability to adapt routines incrementally and change strategies flexibly. For example, in the proactive phase of the first SARS-CoV‑2 wave, some local health boards in the UK took stock of (currently dormant) staff skills and competencies (e.g., doctors and nurses working in management positions). This procedure allowed the immediate redeployment of staff in the event phase (e.g., Aneurin Bevan University Health Board 2020). In this case, adaptability created the capability to implement fast-paced internal communication and rapid decision-making processes to allocate staff wherever needed. In conjunction with fast learning to quickly adapt routines and strategies to changing conditions (Pal *et al.* 2014), this encompassed ***flexibility*** (the core capability of the reactive phase). In this context, flexibility does not utilise the pre-event configuration of services. It enables a health care provider to take whatever resources are available, recombine them, and create new procedures. Structures including feedback loops remain untouched by this adaptive approach.

Conz and Magnani (2020) proposed that, on the micro level, the absorptive and the adaptive resilience paths are equally effective for achieving a positive recovery after an adverse event. On the meso level, their proposition is difficult to verify. On the one hand, resources used to absorb the shock are missing elsewhere. Since an adaptation of the system is less resource-intensive than holding excess supplies in anticipation of a shock, it is plausible to infer that (on the system level) the adaptive approach is more effective than the absorptive one. On the other hand, time and effort to reorganise the allocation and distribution of subjects and objects, adjust processes and make other organisational adaptations are substantial. Moreover, health care budgets are tight, and the benefits of resource use must be carefully balanced—also because public services financed by taxes/social security contributions provide a high proportion of health care funding (71% on average; Organisation for Economic Co-operation and Development [OECD] 2019). Therefore, given how health care organisations currently work, fully operating on the adaptive resilience level (with all stakeholders involved) seems challenging (Schölkopf and Grimmeisen 2021).

Still, there is no doubt that a health care system has to develop and refine absorptive- and adaptive-path capabilities to cope with (small and large) shocks (e.g., hospitals will continue to maintain surge capacities to cope with volatile demand).

We have not yet addressed planned interventions (and, theoretically, the structural adjustments following an adverse event) within the discussion of resilience dimension 4 (see Fig. 1). These interventions intend to elevate the health care system to a new (desired) level of functionality. The health care system needs to change by adapting and adopting innovative technologies, novel models of care and translating them from one health care (sub-)system into another. For example, the Buurtzorg model of nurse-led holistic care that revolutionised community care in the Netherlands (Kreitzer *et al.* 2015) has been successively finding its way into health care systems around the globe (e.g., Lalani *et al.* 2019). When we change the system’s structure (not just incrementally adjust processes), we may reach another (desired) level by planned intervention and likewise when recovering from an adverse event.

#### The Transformative Resilience Path

The transformative phase’s core capabilities are ***efficiency, transformability*** and ***transmutability*** (e.g., Aburn *et al.* 2016; Iflaifel *et al.* 2020; Korber and McNaughton 2018; Turenne *et al.* 2019; Windle 2011).

In its proactive phase, the transformative path entails strategically acquiring the capability of ***efficiency***. Efficiency includes combining and integrating different forms of knowledge (Blanchet *et al.* 2017). For example, health system planners need to understand the resources (currently) available and what is missing (this is the capability of resourcefulness), where (other) weaknesses in the health system lie (conceptually by also geographically), and the expected health care demand. Also, they ought to incorporate the anticipated impact of the legal/regulatory framework and the political context (potentially affecting public health care funding). In this context, all health care managers need to comprehend the impacts of interconnectedness. Another aspect of efficiency is decreasing variation in the system (Lepore and Cohen 1999) to reduce redundancies.

The primary resource of any health care system is its workforce which also accounts for a substantial share of the costs. During the proactive phase, handling volatility and complexity includes sharing the vision (through aims and goals) and bringing clarity. Understanding cause and effect (and sharing the understanding) contributes to creating clarity and makes the system’s ambiguity bearable—the capability to collect and share information further increases the awareness of health care staff. Awareness plus information generates insight. Insight helps staff cope with uncertainty.^1^ With this comprehensive description of *efficiency* in mind, it is plausible to assume that the capability to engage in such a way of handling the system contributes to the capability of health system actors to transform the functions and structure of the health system to (positively) respond to a changing environment (***transformability***) in the event phase. In the response phase, stakeholders can then fundamentally change the health care system to reach a higher (more desirable) level of service delivery (***transmutability***).

In response to the first SARS-CoV‑2 wave, Welsh health boards formed partnerships with the local authorities (hosting local experts familiar with people, sites and local issues). Together, these organisations created exemplary networks successfully carrying out contact tracing during the SARS-CoV‑2 pandemic (Audit Wales 2021). Unlike England, the process was driven by local people (for local people), which united staff and probably affected the public’s compliance. The collaboration went far beyond aligning processes. It created a service with a flat hierarchy, lateral communication flows and a governance structure that involved multi-organisational groups helping with programme management and support offices. With declarations of intent to continue sharing priorities, these networks have the potential capability of reaching a superior level of functionality when it comes to tracing and health protection services (also in a post-SARS-CoV‑2 era). Time will show whether this could serve as a blueprint to generating transmutability, i.e., beneficial structures (not only processes) emerging from the crisis and being utilised for good.

Despite this encouraging example, stakeholders can be very traditional and struggle to engage in the transformative resilience path. For example, after the second SARS-CoV‑2 wave (and before the Delta variant launched the third wave), many health care systems tended to go back to aiming at financial efficiency (instead of operational efficiency). This implied massive unintended consequences. Cutting back on input factors is detrimental for the system in the medium to long run. Enhancing scarcity negatively impacts the timeliness, safety, patient-centredness, effectiveness of care, and, consequently, operational efficiency, which will subsequently decrease financial efficiency. What was needed then and still is needed now is transformation. The transformative capability of the new system would have to enable change (in infrastructure) to reach a higher long-run equilibrium level that is (still) capable of absorbing (further) adverse shocks—*but without unintended consequences.*

**Fig. 6:**
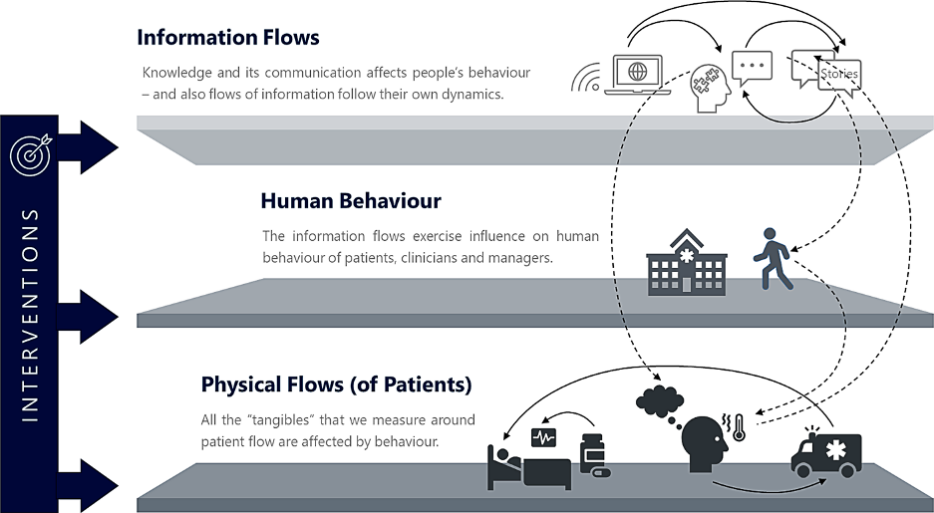
Schematic representation of the layers of a behavioural system dynamics model of health care

A prototypical example of unintended consequences sits at the primary to secondary care interface. British EDs deal with life-threatening conditions, e.g., stroke, breathing difficulties and major trauma such as a road traffic accident (NHS 2021)—at least in theory. In daily practice, patients also come with minor injuries and non-urgent conditions (for which primary care services should have seen them). Patients’ anxiety, misperceived urgency of the presenting complaint, mistaking “urgent” for “life-threatening”, the urge for timely and high-quality emergency care and lack of knowledge about alternative emergency services are some of the reasons non-urgent patients attend an ED (Behrens *et al.* 2021). A way to deal with overwhelmed EDs is to streamline services. Suppose, however, this reaches the limits of what is possible (due to tight inpatient capacities). In that case, the health care system answers with additional resources (e.g., a new emergency care specialist who helps avoid a severe threat to the local population’s health). This intervention reduces waiting times in EDs immediately as staff can now get through a higher number of (urgent and non-urgent) patients. In turn, ED staff gets even more skilled in handling emergency demand. As a result, we have many (urgent and non-urgent) patients who experience an ED as “the place to go” when in need of swift access to diagnostics and specialist treatment—and they talk. Patients share their stories via personal and electronic communication, social media, and their advice for their friends and relatives. The next time someone decides to attend an ED, all these stories come to mind and influence behaviour, choice and eventually patient numbers and flows (see Fig. 6). Also, unintended, the resulting enhancement of an ED’s branding (as a high-quality 24/7 emergency service) creates additional ED demand (patients prefer an ED to primary care emergency services). Hence, with some delay, the positive impact of an additional emergency care specialist is swallowed by the additional ED demand created by a sequence of events kicked off by the (initially positive) intervention. Also, this is resilient system behaviour—just absorptive “resilience” we do not want in this case. The health care system responds favourably in the short run (putting the intervention in the upper right-hand corner in Fig. 2). However, in the medium to long run, the system absorbs the relieving effect of the exogenous intervention by moving demand downstream (from primary to secondary care). This happens via changing the narrative, the “flows of information” that alter patient behaviour and choice (Behrens *et al.* 2021).

What can we learn about handling adverse events from the unintended consequences of a planned intervention?

Interventions (like commissioning additional health care spending) and responses to an adverse event (like asking for compliance with social distances measures) can affect the physical flows of patients either directly or indirectly. A direct effect would be, e.g., building and using a new specialist hospital; indirect effects relate to altering the information flows, which subsequently change behaviour (see Fig. 6). For example, when newspapers and other media report about a (to be built) hospital that provides care with bells and whistles, patients will want to benefit from it. A patient will then choose the specialist hospital over the “traditional” one in a perceived emergency. “*Why shouldn’t I deserve the best possible care?*” The narrative consequently affects behaviour, which then affects the use of health care services (which change the numbers health care managers deal with daily).

Some interventions intentionally focus on changing behaviours (e.g., altering staff conduct such that staff behaviour can serve as a role model during the SARS-CoV‑2 crisis). On a system level, “role modelling” meant that contact tracers worked home-based and not in a physical call centre to send a non-verbal message (“staying at home saves lives”).

Other interventions change behaviour unintentionally (e.g., introducing the 4‑hour target did not achieve that all ED patients are commencing treatment within 240 min; it achieved that staff learned how to stop the patient clock from ticking at 3:59)^2^. The latter implies unintended consequences. In this context and subsequent discussion, *we label a system as resilient if the capacity to absorb, adapt and transform when exposed to an adverse shock will be compatible with responding to positive interventions without displaying unintended consequences*.

Focusing on repairing one element of the health care system can imply counterproductive effects for other parts. Protocols can then call each other out. Therefore, information dissipation needs skilful crafting to avoid undesired behaviours when handling adverse events. The communication of (shared) aims and goals need to be ongoing, and response protocols should be planned in an integrated fashion. The latter needs to incorporate the health care system’s mutual interactions to recover from an adverse event successfully.

### Fifth Resilience Dimension: Characteristics/Capabilities

**Fig. 7:**
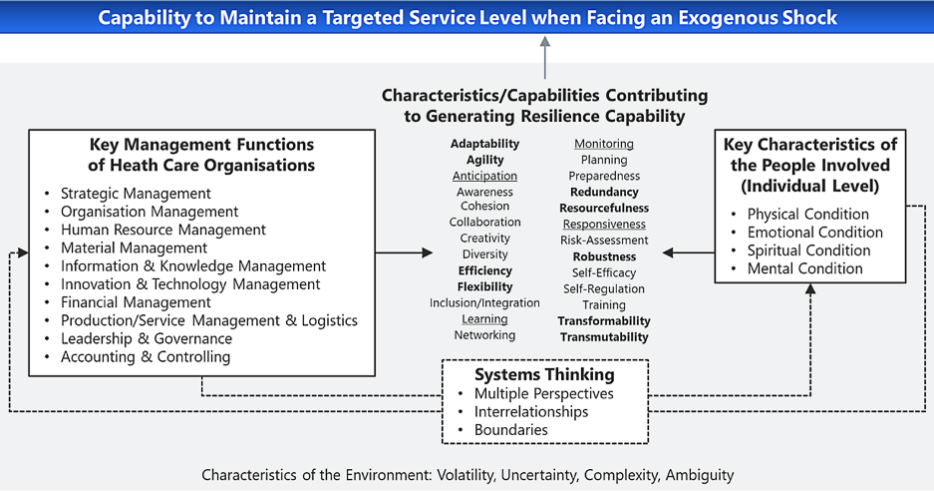
Resilience dimension 5: organisational and individual characteristics and capabilities to generate resilience capability of the health care system (developed based on Bea and Haas 2019; Hollnagel 2011; Paulsen and Hernes 2003; Windle 2011; Wolstenholme and McKelvie 2019 and general resilience review literature)

The fifth resilience dimension puts into context the characteristics and additional capabilities identified in the literature as essential to overcome an adverse event (e.g., the SARS-CoV‑2 pandemic) or support a planned intervention (e.g., a health care reform). The aim is to reach or maintain a targeted service level. The fifth dimension consists of the following four essential components (see Fig. 7): 1) key characteristics of people involved (cf. Sect. 3.5.1), 2) key management functions of health care organisations (cf. Sect. 3.5.2), 3) systems thinking (cf. Sect. 3.5.3) and 4) characteristics/capabilities contributing to generating resilience capability (cf. Sect. 3.5.4). Please note that the characteristics of the environment (cf. Sect. 3.2), which are volatile, uncertain, complex and ambiguous (cf. Bennett and Lemoine 2014), should be incorporated in all four components.

#### Key Characteristics of the People Involved

The resilience of people, including health care staff (e.g., Palacio Gonzalez *et al.* 2020) and patients (e.g., Kim *et al.* 2019), in an event phase depends on pre-existing physical, emotional, spiritual and mental conditions (and on fostering and sustaining them; see the right-hand box in Fig. 7). These conditions respond to aptitudes, behaviours, motivation and temperament. Furthermore, people are rooted in families, households, neighbourhoods (Windle 2011) and other environments (see Fig. 3). Thus, fostering and reconstituting the resilience of health care professionals (the critical input factor of the health care sector), including their physical, emotional, spiritual and mental health, is of utmost importance for system resilience (e.g., Palacio Gonzalez *et al.* 2020), especially in times of the SARS-CoV‑2 pandemic (e.g., Labrague 2021; Rieckert *et al.* 2021; Zhang *et al.* 2022).

For example, Heath *et al.* (2020) outlined that health care professionals’ workload and stress have been enormous during the SARS-CoV‑2 pandemic. It might take years to recover and re-establish resilience. At the same time, some health care workers might leave the sector due to compulsory SARS-CoV‑2 pandemic vaccinations, which worsens the already critical situation of health care organisations (Biswas *et al.* 2021). Furthermore, especially patients with chronic diseases suffered from general restrictions and access problems to health care services during the SARS-CoV‑2 pandemic (e.g., Vázquez *et al.* 2021). Therefore, an adverse event (e.g., a pandemic) requires broad interventions to foster resilience at the individual level (e.g., increasing engagement in self-care and mindfulness), organisational level (e.g., offering wellbeing initiatives), community level (e.g., increasing social capital of communities such as prosocial behaviour of helping others) and the national level (e.g., cultivating resilient leadership).

#### Key Management Functions of Health Care Organisations

Health care staff are embedded in health care organisations whose management, that is, individual (groups of) decision-makers, holds essential management functions (see the left-hand box in Fig. 7). These include strategic management, organisational management, human resource management, material, information & knowledge management, innovation & technology management, financial management, production/service management & logistics, leadership & governance and accounting & controlling (e.g., Bea and Haas 2019).

Satisfactory management functions are the result of appropriate characteristics/capabilities. The list of resilience-relevant characteristics and capabilities of organisations and workforce identified in the literature is long (e.g., Barasa *et al.* 2017; Conz and Magnani 2020; Curt and Tacnet 2018; Fridell *et al.* 2020; Kruk *et al.* 2017; Rahi 2019; Windle 2011). Still, it is not about acquiring these characteristics/capabilities one by one to somehow engender resilience magically. Instead, these characteristics/capabilities are the by-products of the iterative (dynamic) process sketched in Fig. 5. The resulting meshwork of interconnected and mutually reinforcing characteristics/capabilities “create” the system’s capability to reach/maintain a targeted level of service after an adverse shock (or achieve the desired purpose level through a planned intervention). The system thinking approach, which understands the health care system as a complex aggregate of mutually influencing subsystems, appropriately links the functions and characteristics/capabilities to the resilience concept.

Hollnagel (2011) identifies the capabilities of 1) learning, 2) responding, 3) monitoring and 4) anticipating (underlined in Fig. 7) as the four cornerstones for building resilience capacity at the system level. They neatly fit into the framework presented so far and enable actors to know what has happened (capability of learning), to know what to do (capability of responding), to know what to look for (capability of monitoring) and to know what to expect (capability of anticipating).

Including this context information, Fig. 7 supplies policymakers with a rationale for the prerequisites to reach or maintain a targeted level of service delivery. Patient-centredness, safety, equity, efficiency, effectiveness and timeliness of care are multi-criteria quality domains (Institute of Medicine 2001) that health care policymakers can use to evaluate whether they have come sufficiently close to the desired target level services.

The way the capabilities are interconnected and imply each other leads back to resilience dimensions 3 and, particularly, 4. For example, monitoring enables the capability of anticipating. Anticipation elevates awareness. Full awareness of the current situation plus anticipating the future (and the capability of assessing risks) allow planning. The capability to plan allows preparing for the future, e.g., it kicks off the absorptive and adaptive resilience paths (see Fig. 4), yielding the capability to respond to exogenous events. (Note that the core capabilities along the three resilience paths are bolded in Fig. 7 for faster identification.) Responses generate learning which feeds back into the capabilities of anticipating and planning. The management style and workforce involved should be creative, agile, inclusive and flexible. Integration, cohesion, collaboration, diversity (including diversity of thought) and networking are further resilience drivers among internal and external stakeholders. Organisations with self-regulation and a workforce accumulating self-efficacy can better withstand adverse effects (especially when moving along the transformative resilience path).

#### Systems Thinking

Health care organisations and people (i.e., individual workforce members and patients) are interconnected (cf. resilience dimension 2 in Fig. 3). They can thus directly contribute to the emergence of the capabilities nurturing and cultivating resilience or indirectly affect other parts of the system to do so. In particular, organisations cannot “generate resilience capability” by merely adjusting procedures but not incorporating staff. Likewise, it is utopian to assume that people can consistently step in where the system fails. Thus, whatever happens in one domain reinforces what happens in another domain of the interconnected system sketched in Fig. 7. We signify this by the Systems Thinking box governing the influence of human characteristics on organisational functions and *vice versa*. Acknowledging multiple perspectives, appreciating interconnectedness (Wolstenholme and McKelvie 2019) and especially successfully managing boundaries (Paulsen and Hernes 2003) are key to positive mutual reinforcement.

Throughout this paper, we have given several examples of the importance of Systems Thinking for health care planning. They included the discussion of stakeholders in the context of virtual clinics (in Sect. 3.2), the importance of aligning stakeholder/organisation by shared aims and priorities (in Sect. 3.2), the opportunities arising from flexible redeploying staff (in Sect. 3.4) and the importance of knowing the relationship among patient and information flows, behaviours and interventions for service redesign in Sect. (3.4).

## Conclusions and Policy Implications

This paper identified, discussed and augmented critical dimensions of resilience retrieved from the academic literature. In this context, we took a closer look at the impact of complexity and behaviour change prompted by planned interventions in the health care sector alongside management of adverse events. We found that a system’s ability to follow absorptive, adaptive and transformative resilience paths simultaneously enables the system to cope with adverse events *and* respond to planned interventions without unintended negative consequences annihilating desirable outcomes. “Knowing your system (well)” is a prerequisite for developing *redundancy, resourcefulness* and *efficiency* capabilities (the entry points of the three resilience paths discussed in this paper). This requirement pertains to managing the system as a whole instead of an accumulation of parts. Active interface management to align stakeholder priorities with overarching purposes becomes crucial—especially for collaborating across organisations in a complex health care system.

Still, most resilience literature focuses on dealing with adverse events and, thus, absorptive resilience. For some adverse events, however, we also observe adaptive resilience efforts. The management capabilities to follow adaptive resilience would also lend themselves to focus on (incremental) process improvement. However, we rarely see a health care system recovering from an adverse event through transformative resilience, which alludes to structural changes alongside process adaptation.

Our central finding in the context of resilience in health care is the necessity to acquire the right mix of management capabilities to deal with exogenous shocks. We highlighted that this corresponds to the necessity of adapting strategies to build health care resilience that accommodate both the response to adverse events and planned interventions.

It becomes apparent only when dealing with planned interventions that transformative capabilities are indispensable to cope with sudden increases in health care pressures. This is because absorbing adverse effects and adapting processes to restore pre-shock levels do not necessarily require transformation. Therefore, organisations may be inclined to over-rely on fostering capabilities necessary for absorptive and adaptive resilience (and go around wrangling the beast of acquiring transformative capabilities). Therefore, the focus on absorption and adaptation is counterproductive for dealing with adverse events because the health care system and its stakeholders are misled to focus on short-term fixes, not sustainable change. In other words, focusing the resilience discussion only on adverse events could deceive organisations into cultivating a suboptimal mix of organisational capabilities lacking transformative capabilities, which pave the way for structural changes that aim at a sustainably higher functionality.

We underpin our claim in the context of the SARS-CoV‑2 pandemic, for which we observed various system responses and interventions along the absorptive and adaptive paths. Transformative interventions strategically made towards a structural change of the health care system to increase its functionality beyond the pre-shock level are scarce. Sadly, most of the rare cases where the potential for system transformation was generated out of the crisis started to rapidly wither away because health care organisations do not (yet) have cultivated the capabilities to maintain these structures in the long run.

Coping with the pandemic, *redundancies* like an inventory full of personal protective equipment, an excess amount of hospital beds, a backup team of adequately trained staff and an inventory filled with medical instruments and pharmaceutical drugs enabled the health care system/providers to face the demand shock and gradually recover from the first wave in spring 2020. For example, due to the high bed capacity rate (7.2 acute beds/1000 inhabitants) and a strict lockdown policy in mid-March 2020, Austria surmounted the first wave of the SARS-CoV‑2 pandemic without triaging and spikes in excess mortality. At the same time, countries like Italy, Spain and the UK (with less than half of Austria’s hospital bed capacity per 100,000 inhabitants) were hit badly (OECD 2022). Countries with low acute bed capacities had to establish new treatment facilities such as field hospitals, as Haldane et al. (2021) reported. Others (additionally) relied on home-based health care strategies for patients with mild or moderate symptoms (Haldane *et al.* 2021) and various lockdown strategies to create and ensure sufficient hospital capacity. However, the strategies varied from no, moderate, strict lockdowns to zero-SARS-CoV‑2-measures at all, depending on demographics, politics, geographical location, culture and tradition (Steffen 2022). Public health interventions like isolation, quarantine, contact tracing, hygiene, masks, social distancing, testing, school/university closures and distance learning also played a significant role at the pandemic’s beginning (Iezadi *et al.* 2020).

Altogether, we increasingly observe such-like adaptive interventions from wave 1 onwards (spring 2020). Moving along the adaptive resilience path enabled health care services to take stock of staff and redeploy them wherever needed. Many health care providers also adapted and adopted digital technologies and increasingly provided telehealth services (e.g., digital prescription), as outlined by Haldane *et al.* (2021). Countries consistently adapted their testing strategies and capacities to current demand depending on the budget. Also, they prioritised vulnerable groups or critical infrastructure providers for scarce testing (Haldane *et al.* 2021).

Several Northern Hemisphere countries employed recurring lockdown strategies in autumn 2020 as other public health interventions could barely curb the reproduction rate of this airborne virus below one (Steffen 2022). By the end of 2020, vaccinations also started to positively contribute to the pandemic response (at least to some extent). Zheng *et al.* (2022), performing a meta-analysis concerning the effectiveness of SARS-CoV‑2 vaccines for fully vaccinated adults, found an infection protection rate of more than 90%. However, these figures sharply dropped with the infectious and rather severe Delta variant (summer/autumn 2021) and the even more infectious but less severe Omicron variant (winter 2021/22) (Zheng *et al.* 2022). Given the reports of the more than one million vaccinated persons with side effects documented by the European Medicines Agency (EMA 2022) and their amplification via social media, vaccination campaigns could not get enough traction. Nehal *et al.* (2021), for example, estimated the global SARS-CoV‑2 vaccination willingness at about 66% in 2021.

As of autumn 2021, the first-line strategy was a booster vaccination, protecting people from severe illness and hospitalisation at about 96% and 79% for the Delta and Omicron variants, respectively (Hogan *et al.* 2022). As the booster vaccination uptake was insufficient to flatten the curve to the desirable extent, other lockdowns came into place in autumn 2021 and winter 2021/22, mainly to create *redundant* hospital resources (Steffen 2022). Some might be inclined to call that “back to square one”. However, the Omicron variant (currently leading to high infection rates in many countries) might disappear before an effective vaccine is available, as Waltz (2022) noted. One scenario even assumes that Omicron will increase the population’s immunity to further SARS-CoV‑2 variants so that the epidemic can be managed with influenza-like strategies from autumn 2022 onward (Waltz 2022). These strategies may allow deviating from a general compulsory vaccination strategy and punishment of people not (fully) vaccinated; measures that divide the people and lead to demonstrations worldwide.

None of these counter-SARS-CoV‑2 measures goes down the transformative path. None of it includes the anticipated health care pressures due to long COVID and increased number of mental disorders that will keep health care systems busy over decades (Rivas-Vazquez *et al.* 2022). None of them factors in potential side effects of vaccinations (EMA 2022). All we have observed is one short-term fix that follows the preceding one, showing another danger of over-relying on absorptive and adaptive resilience capabilities. These interventions work once (or twice), but they are not designed to deal with an ongoing chain of adverse events. This is why we face depleted health care systems and an exhausted workforce in nearly all health care systems around the globe (Heath *et al.* 2020). One may wonder that—if applying systems thinking in health care could pay off so nicely—what have been the barriers to using it. Why do we not integrate absorptive, adaptive and transformative resilience capabilities to reach superior levels of functionality consistently?

Firstly, aligning stakeholder priorities to serve an overall purpose is challenging. Even facing a pandemic is characterised by local objectives and goals (like increasing local vaccination rates). Health care stakeholders promote boundaries to manage their domains optimally. However, optimising one sector often comes at the expense of another. Tackling this problem is complex because the areas of responsibility are not subject to stakeholder choice but are often constitutionally regulated. Also, attempts to alter the stakeholders’ competencies often cause considerable resistance. For example, the merger of the regional statutory health insurance companies into a single Austrian organisation in 2020 generated the opposition of the insurance representatives, who insisted that their planning and contribution sovereignty prevail.

Secondly, service providers, e.g., physicians, and recipients, i.e., patients, aggravate interface problems. Just as health care providers are not necessarily interested in harmonising reimbursement, neither are (all) service recipients interested in reconciling services.

Thirdly, we often observe delays when implementing health care measures. By separating cause and effect, delays compromise the human ability to distinguish what measures actually solved (or aggravated) a problem. In the presence of many corrective actions, we, additionally, fall short of the competence to pin down the extent of a single measure’s effect. Additionally, corrective measures can become an ongoing (and long-lasting) process where we can quickly lose sight of the original intervention’s primary purpose.

In a nutshell, building resilient systems needs to be strategically approached, not leaving it with the grassroots and exploiting individual passion and engagement. It needs to be built on the integrated presence of the capabilities characterising the absorptive, adaptive and transformative resilience paths. Therefore, systems thinking must be taken on board, acknowledging that health care systems are interconnected and dynamic systems that include feedback. Further cross-relationships with other systems exist in this context and must also be incorporated to build a resilient health care system. For example, the educational and health systems are closely related to providing a suitably trained workforce when needed. It has to acknowledge that information, behaviours and physical measures (like patient numbers) are intertwined and must be integrated into resilience design. Finally, we must design health care systems around patient needs, not provider convenience. Who works together needs aligned goals and synchronised priorities. The (single) voice of the patient can unite the multiple voices of the stakeholders and engineer one system to serve cost-efficiently (and resiliently) at the right time by the right person at the right place.

## Footnotes

1 See Bennett and Lemoine (2014) for a succinct overview of what volatility, uncertainty, complexity and ambiguity (VUCA) can “*mean for you*”.

2 We observe that patients are transferred from the ED to the Medical Assessment Unit (MAU) towards the end of the 4‑hour time window because a MAU has no target throughput time. At the front end, we observe a reluctance to admit patients who arrive via ambulance. This leads to ambulance cars queueing outside hospitals—not releasing this vital resource needed by other patients. In this context it is worth mentioning that unintended behaviour change sometimes originates from “unintended interventions”. A typical example is to use descriptive data for performance management (Solberg *et al.* 1997). Staff responds differently to knowing that an ED patient waited 4.5 h until commencing treatment and to knowing that they will be told off for it.
